# Polymerization kinetics of experimental resin composites functionalized with conventional (45S5) and a customized low-sodium fluoride-containing bioactive glass

**DOI:** 10.1038/s41598-021-00774-w

**Published:** 2021-10-27

**Authors:** Matej Par, Katica Prskalo, Tobias T. Tauböck, Hrvoje Skenderovic, Thomas Attin, Zrinka Tarle

**Affiliations:** 1 Department of Endodontics and Restorative Dentistry, School of Dental Medicine, University of Zagreb, Gunduliceva 5, Zagreb, Croatia; 2 Department of Conservative and Preventive Dentistry, Centre for Dental Medicine, University of Zurich, Plattenstrasse 11, Zurich, Switzerland; 3 Institute of Physics, Bijenicka cesta 46, Zagreb, Croatia

**Keywords:** Composite resin, Dental materials, Dental biomaterials

## Abstract

This study aimed to investigate polymerization kinetics and curing light transmittance of two series of experimental dental resin composites filled with 0–40 wt% of either 45S5 bioactive glass (BG) or a customized low-Na F-containing BG. Polymerization kinetics in 0.1-mm and 2-mm thick layers were investigated through real-time degree of conversion measurements using a Fourier transform infrared (FTIR) spectrometer. FTIR spectra were continuously collected at a rate of 2 s^−1^ during light-curing (1340 mW/cm^2^). Light transmittance through 2-mm thick composite specimens was measured using a UV–Vis spectrometer at a rate of 20 s^−1^. Unlike BG 45S5, which led to a dose-dependent reduction in the rate and extent of polymerization, the customized low-Na F-containing BG showed a negligible influence on polymerization. The reduction in light transmittance of experimental composites due to the addition of the low-Na F-containing BG did not translate into impaired polymerization kinetics. Additionally, the comparison of polymerization kinetics between 0.1-mm and 2-mm thick layers revealed that polymerization inhibition identified for BG 45S5 was not mediated by an impaired light transmittance, indicating a direct effect of BG 45S5 on polymerization reaction. A customized low-Na F-containing BG showed favourable behaviour for being used as a functional filler in light-curing dental resin composites.

## Introduction

Dental resin composites represent a versatile material class for various dental applications, including restorative dentistry, orthodontics, prosthodontics, pedodontics, endodontics, and preventive dentistry. Notwithstanding experimental composites developed for research purposes, most contemporary dental composites used in daily clinical practice are based on mixtures of multifunctional methacrylate resins filled with silanized glass/silica/zirconia particles of heterogeneous sizes^1^. The composites are rendered photosensitive using blue light-absorbing compounds, which makes them capable of on-demand setting through free radical-mediated polymerization of methacrylate groups.

The formation of the polymer network during the setting of resin composites is a complex reaction which occurs in a medium of rapidly increasing viscosity. The mobility of reactive species declines as polymerization advances, leading to complex crosslinked networks that eventually become immobilized, hindering further polymerization and leaving a fraction of C=C double bonds unreacted^2^. The fundamental parameter used for evaluating the extent of the polymerization is the degree of conversion, and real-time monitoring of this parameter allows for the characterization of polymerization kinetics^3^.

The fact that secondary caries represents the primary cause for long-term failures of otherwise successful composite restorations has motivated extensive investigations of various reactive additives that could exert a protective effect on dental hard tissues^4^. Some of the protective mechanisms include discouraging the development of bacteria and biofilm formation on the tooth surface^5^, changing the pH and ion concentrations to inhibit demineralization and promote remineralization of dental hard tissues^6,7^, and improving the strength and sealing of the adhesive interface between the tooth cavity and restoration^8,9^, thus discouraging bacterial accumulation within marginal flaws^10^. Among various reactive additives for resin composites, bioactive glasses (BG) are especially interesting due to their capability to exert practically all of the aforementioned beneficial effects^11–13^.

As BG compositions can be conveniently adjusted by changing the amounts of reactants in the production process^14^, various customized BG types have been investigated as potential reactive fillers for dental resin composites^11,15,16^. A series of studies on experimental composites indicated that the addition of BG 45S5 to experimental composites can induce a dose-dependent inhibition of methacrylate polymerization^17–20^. This effect was manifested as a lower final extent of polymerization reaction^17–19^, as well as a slower polymerization rate^20^. As a potential alternative for BG 45S5, a customized low-Na F-containing BG has been developed which is characterized by better stability and capability to release fluoride^21–23^. A series of studies have shown its favourable properties, namely acid neutralization and hydroxyapatite precipitation^21^, favourable curing properties^22^, and protective effect on enamel^23^.

This study investigated the polymerization kinetics of two series of experimental composites filled with 0–40 wt% of either BG 45S5 or a customized low-Na F-containing BG. As an important determining factor for light-activated polymerization^24^, light transmittance through the composite materials was additionally investigated. The null hypotheses assumed that (I) polymerization kinetics of experimental composites would not be affected by BG type, BG amount, and composite layer thickness; and (II) that light transmittance through the clinically representative layer thickness (2 mm) would not be affected by BG type and BG amount.

## Materials and methods

### Experimental resin composites

The composition of fillers used in the preparation of experimental composites is shown in Table 1. A customized low-Na F-containing experimental BG with theoretical network connectivity similar to that of BG 45S5 (2.1) was prepared on-demand by Schott (Mainz, Germany) via a melt-quench route. The melt-quench route was selected as a cost-effective method that is more convenient for incorporating fluorides compared to the sol–gel technique^11^. Other fillers (BG 45S5, inert barium glass, and silica) were obtained from commercial vendors. Similar preparation and grinding procedures were used to ensure comparable particle sizes of the F-containing BG and BG 45S5.

**Table 1:** Bioactive glass and reinforcing fillers used in experimental composites.

	Bioactive glass 45S5	Experimental fluoride-containing bioactive glass	Inert barium glass	Silica
Particle size (d50)	3 µm	3 µm	1 µm	5–50 nm
Composition (wt%)	45.0% SiO_2_ 24.5% CaO 24.5% Na_2_O 6.0% P_2_O_5_	33.5% SiO_2_ 33.0% CaO 10.5% Na_2_O 11.0% P_2_O_5_ 12.0% CaF_2_	55.0% SiO_2_ 25.0% BaO 10.0% Al_2_O_3_ 10.0% B_2_O_3_	> 99.8% SiO_2_
Silanization (wt%)	None	None	3.2	4–6
Manufacturer	Schott, Mainz, Germany	Schott, Mainz, Germany	Schott, Mainz, Germany	Evonik, Hanau, Germany
Product name/LOT	G018-144/M111473	experimental batch	GM27884/Sil13696	Aerosil R 7200/157020635

The resin system consisted of a 60:40 wt% mixture of bisphenol-A-glycidyldimethacrylate (Bis-GMA; Merck, Darmstadt, Germany) and triethylene glycol dimethacrylate (TEGDMA; Merck). Camphorquinone (0.2 wt%; Merck) and ethyl-4-(dimethylamino) benzoate (0.8 wt%; Merck) were added as a photoinitiator system^25^. The monomers and photoinitiators were blended in a dark container using a magnetic stirrer for 48 h.

The experimental composites with a total filler load of 70 wt% and varying amounts of BG (0–40 wt%) were prepared following previous studies^21,23^. The detailed composition of experimental composites is given in Table 2. The composite containing only reinforcing fillers (0 wt% of BG) was used as a control. The composites were prepared by mixing the photoactivated Bis-GMA/TEGDMA resin mixture and the fillers for 5 min in a centrifugal mixer (Speed Mixer TM DAC 150 FVZ, Hauschild & Co. KG, Hamm, Germany) at 2000 rpm, followed by deaerating in vacuum for 48 h.

**Table 2:** Composition of experimental composites.

Material designation	Filler composition (wt%)			Total filler ratio (wt%)
	Bioactive glass 45S5	Low-sodium fluoride-containing bioactive glass	Reinforcing fillers (inert barium glass: silica = 2:1)	
Control	0	0	70	70
**C-series**				
C-5	5	0	65	70
C-10	10	0	60	70
C-20	20	0	50	70
C-40	40	0	30	70
**F-series**				
F-5	0	5	65	70
F-10	0	10	60	70
F-20	0	20	50	70
F-40	0	40	30	70

**Figure 1:**
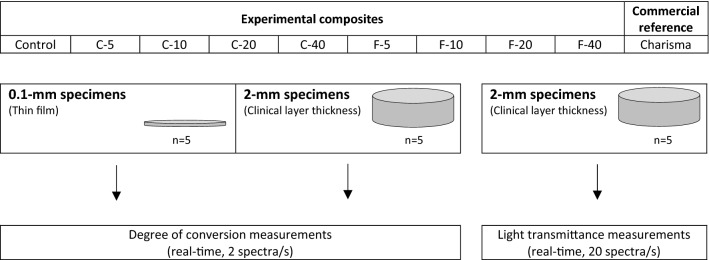
Experimental design.

In addition to the experimental composites, a commercial Bis-GMA/TEGDMA-based restorative composite (Charisma Classic; Kulzer, Hanau, Germany; LOT: K010752, EXP: 2024-03-17) was used as a reference. A schematic summary of the study design is given in Fig. 1.

### Degree of conversion

The polymerization kinetics were investigated through real-time degree of conversion (DC) measurements using a Fourier transform infrared (FTIR) spectrometer (Nicolet iS50, Thermo Fisher, Madison, USA) with an attenuated total reflectance (ATR) accessory. Uncured composites were placed on the diamond ATR crystal using Teflon moulds to obtain discoid specimens (diameter = 6 mm thickness = 0.1 or 2 mm). The composite specimens were covered with polyethylene terephthalate (PET) strips and light-cured for 20 s using Bluephase PowerCure (Ivoclar Vivadent, Schaan, Liechtenstein). As measured using a calibrated and NIST-referenced UV–Vis spectrophotometer system (MARC; BlueLight Analytics, Halifax, Canada), the radiant exitance of the curing unit was 1340 mW/cm^2^. The curing unit was positioned perpendicularly and in direct contact with the composite specimen surface. FTIR spectra were continuously collected at a rate of 2 spectra per second for 5 min after the start of light-activated curing, with 4 scans and a resolution of 8 cm^−1^. Five specimens per experimental group were tested (n = 5).

DC was calculated from the changes in the ratio of absorbance intensities of the spectral bands at 1638 cm^−1^ (aliphatic C=C) and 1608 cm^−1^ (aromatic C⋯C), using the following equation^26^:

$$DC \left( \% \right) = \left[ {1 - \frac{{\left( {1638\; {\text{cm}}^{ - 1} /1608 \;{\text{cm}}^{ - 1} } \right)_{peak\; height\; after \;curing} }}{{\left( {1638\; {\text{cm}}^{ - 1} /1608 \;{\text{cm}}^{ - 1} } \right)_{peak \;height \;before\; curing} }}} \right] \times 100.$$

The DC data were plotted as a function of time and first derivatives were calculated to represent the reaction rate^27^. The obtained reaction rate was plotted as a function of time to determine the maximum reaction rate (R_max_) and time to reach maximum reaction rate (t_max_). Additionally, the DC values reached at the end of the 5 min observation period (DC_5 min_) were evaluated.

The curves of DC versus time were fitted by a four-parameter exponential sum function.

y = a*(1 − e^−bx^) + c*(1 − e^−dx^)^27^. The four modulation parameters in this equation are used to describe polymerization kinetics during the gel phase (parameters a and b) and the glass phase (parameters c and d)^3,28^.

## Light transmittance

Light transmittance through 2-mm composite specimens was measured in real-time during light-curing with 1340 mW/cm^2^ for 20 s using Bluephase PowerCure. Composite specimens (diameter = 6 mm, thickness = 2 mm) were prepared in black Teflon moulds and sandwiched between two PET strips that covered mould openings. Five specimens per experimental group were tested (n = 5). Visible light that passed through composite specimens was collected using a lens and directed into a charge-coupled device array fibre spectrometer HR4000 (Ocean Optics, Dunedin, FL, USA) and spectra were collected at a rate of 20 s^−1^. In the same manner, visible light spectra of the empty specimen compartment were recorded. Relative light transmittance (%) based on the integrated intensity over the whole wavelength range emitted by the curing unit (380–520 nm) was calculated as the following ratio^29^:

$$Light\; transmittance\; \left( \% \right) = \frac{{Light\; intensity\; \left( {composite \;specimen} \right)}}{{Light\; intensity\; \left( {empty\; specimen \;compartment} \right)}} \times 100$$

Light transmittance values were calculated for each spectrum collected through real-time measurements over the 20-s light-curing period. As light transmittance gradually increased during light-curing due to the increasing refractive index of the resin which undergoes the transition from monomer into polymer^29^, the “effective” light transmittance, trans_ef_, was calculated by integrating light intensity curves for the whole observation period and dividing the obtained mathematical areas for composite specimen by the corresponding areas obtained while recording curing light spectra through the empty specimen compartment^19^. The parameter trans_ef_ calculated in this manner is a useful simplification as a single light transmittance value is representative for the whole curing period, i.e., multiplying that value by the irradiance received by the specimen gives the total radiant exposure at the composite specimen bottom.

### Statistical analysis

Normality of distribution was evaluated using Shapiro–Wilk’s test and inspection of normal Q–Q plots. Levene’s test was used to verify the homogeneity of variances. The variables DC_5 min_, R_max_, and t_max_ were compared among the composites within a given layer thickness (0.1 or 2 mm) using one-way ANOVA and Tukey’s adjustment for multiple comparisons. The trans_ef_ values were compared among the materials using one-way ANOVA with Tukey’s post-hoc adjustment. T-test for independent observations was used for pairwise comparisons between 0.1-mm and 2-mm thick layers for DC_5 min_, R_max_, and t_max_. For the fit parameters of the double exponential sum function (a, b, c, and d), the 95% confidence intervals were calculated and used to identify significant effects of variations in the BG amount within the experimental composite series. The statistical analysis was performed using SPSS (version 25; IBM, Armonk, NY, USA) at an overall α = 0.05.

## Results

**Figure 2:**
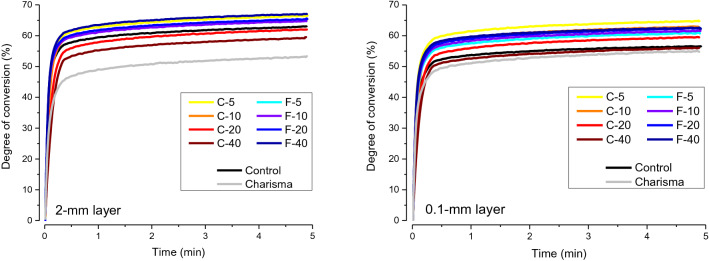
Averaged curves (n = 5) of degree of conversion at the bottom of 2-mm and 1-mm layers as a function of time.

**Figure 3:**
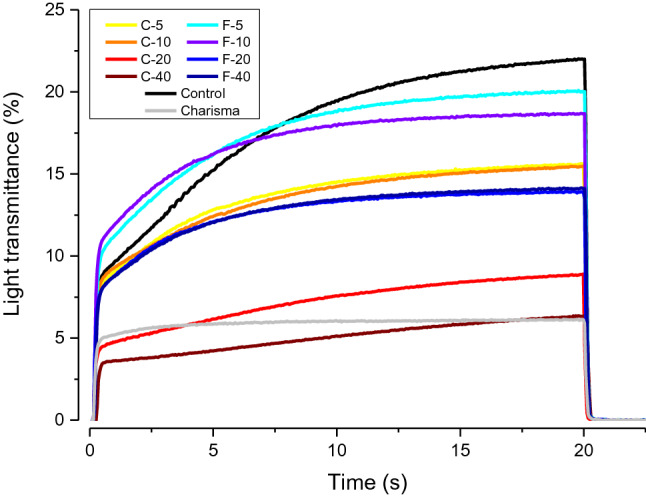
Averaged curves (n = 5) of light transmittance through 2-mm composite layers as a function of time.

The real-time DC and light transmittance curves are illustrated in Figs. 2 and 3, respectively. The DC curves shown in Fig. 2 were used to determine DC_5 min_, R_max_, and t_max_. The light transmittance curves shown in Fig. 3 were used to calculate the trans_ef_.

**Figure 4:**
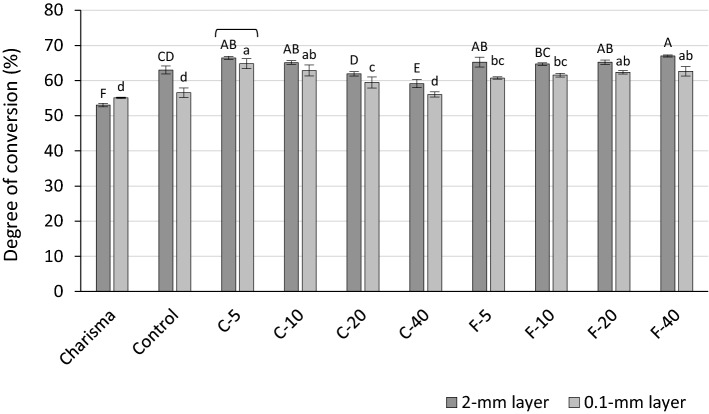
Degree of conversion (mean values ± standard deviations) measured after 5 min. Statistically homogeneous groups are indicated by same uppercase letters for 2-mm layers and same lowercase letters for 0.1-mm layers. Square brackets indicate statistically similar values for comparisons of 2 mm versus 0.1 mm.

DC_5 min_ is presented in Fig. 4 and ranges from 53.0 to 67.0%. DC_5 min_ was significantly higher for the 2-mm layers than 0.1-mm layers, except for Charisma (inverse relationship) and C-5 (statistically similar values). For both layer thicknesses within the C-series, a statistically significant DC_5 min_ decrease was identified as a result of increasing BG amounts. In contrast, DC_5 min_ values in the F-series were mostly statistically similar.

**Figure 5:**
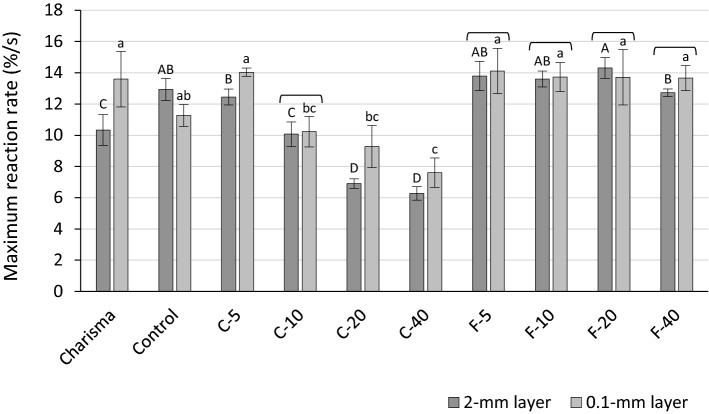
Maximum polymerization reaction rate (mean values ± standard deviations). Statistically homogeneous groups are indicated by same uppercase letters for 2-mm layers and same lowercase letters for 0.1-mm layers. Square brackets indicate statistically similar values for comparisons of 2 mm versus 0.1 mm.

**Figure 6:**
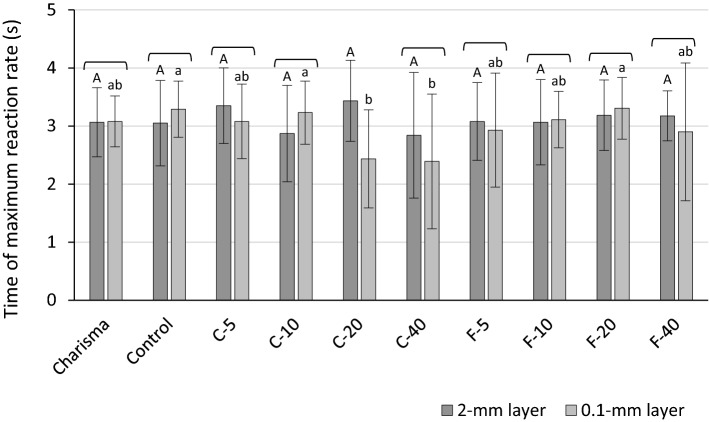
Time to achieve maximum polymerization rate (mean values ± standard deviations). Statistically homogeneous groups are indicated by same uppercase letters for 2-mm layers and same lowercase letters for 0.1-mm layers. Square brackets indicate statistically similar values for comparisons of 2 mm versus 0.1 mm.

R_max_ is shown in Fig. 5. The effect of layer thickness was statistically significant in the C-series, identified as a higher polymerization rate in 0.1-mm than in 2-mm layers. In contrast, F-series showed statistically similar R_max_ regardless of the layer thickness. Whereas for C-series increasing BG amounts resulted in the significant decrease in R_max_ at both layer thicknesses, the F-series showed no systematic changes in R_max_ as a function of BG amount.

t_max_ presented in Fig. 6 showed high coefficients of variability (14–49%), thus being less discriminative in statistical comparisons. The statistically significant effect of layer thickness was identified only for C-20, while all other materials showed similar values regardless of the layer thickness. Inter-material comparisons showed that t_max_ values were mostly statistically similar.

**Figure 7:**
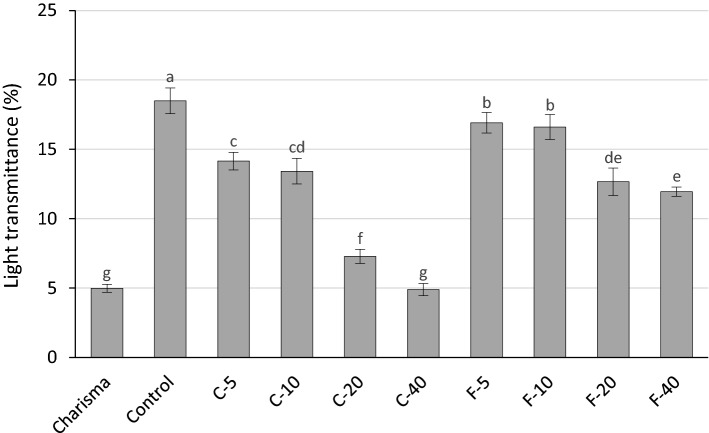
Effective light transmittance values (mean values ± standard deviations) for 2-mm layers. Statistically homogeneous groups are indicated by same lowercase letters.

Figure 7 represents trans_ef_ through 2-mm composite layers. All experimental composites except C-40 showed significantly higher light transmittance than the reference composite (Charisma). For both the C-series and F-series, light transmittance was significantly diminished by increasing BG amounts. For a given BG amount, the composites from the F-series had higher light transmittance than the corresponding composites of the C-series.

**Figure 8:**
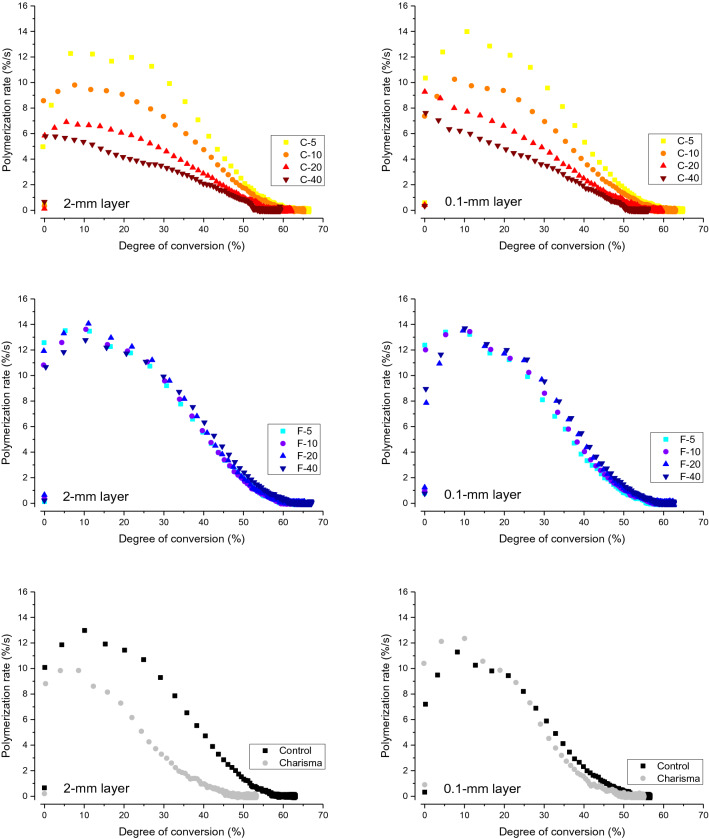
Polymerization rate as a function of degree of conversion (averaged data for n = 5).

Polymerization rate as a function of DC is plotted in Fig. 8. In the F-series, the polymerization rates peaked at DC values 8–12% with no systematic effect of the BG amount. In contrast, polymerization rate maxima in the C-series were gradually shifted towards lower DC values as the BG amount increased.

The fit parameters of the exponential sum function y = a*(1 − e^−bx^) + c*(1 − e^−dx^) are presented in Table 3 (0.1-mm layer) and Table 4 (2-mm layer). In the C-series, the gel phase parameters “a” and “b” were significantly reduced by higher BG amounts. The glass phase parameter “c” was significantly reduced by higher BG amounts only in the 0.1-mm layer, while the reduction of the glass phase parameter “d” was identified for both layer thicknesses. In the F-series, the parameter “a” was significantly increased by higher BG amounts, while no significant effect was observed for the parameter “b”. The parameter “c” in the F-series was significantly reduced by increasing BG amounts only for the 0.1-mm layer, while the parameter “d” was unaffected at both layer thicknesses.

**Table 3:** Fit parameters of exponential function (95% confidence interval in parentheses) for 0.1-mm layer.

	a	b	c	d
Control	56.61 (55.71–57.51)	15.36 (14.94–15.78)	6.44 (6.17–6.71)	0.77 (0.69–0.84)
C-5	65.83 (64.67–66.99)	17.69 (17.18–18.21)	7.66 (7.33–7.98)	0.82 (0.74–0.89)
C-10	63.82 (63.14–64.51)	13.61 (13.36–13.78)	6.96 (6.77–7.16)	0.63 (0.58–0.68)
C-20	57.81 (57.49–58.13)	11.23 (11.12–11.34)	6.86 (6.76–6.96)	0.56 (0.53–0.59)
C-40	55.32 (54.99–55.66)	9.73 (9.63–9.84)	6.40 (6.25–6.56)	0.38 (0.34–0.41)
F-5	60.10 (59.03–61.16)	17.29 (16.78–17.79)	7.38 (7.10–7.67)	0.76 (0.69–0.83)
F-10	61.17 (60.05–62.29)	17.53 (17.00–18.06)	7.32 (7.02–7.63)	0.80 (0.72–0.87)
F-20	63.92 (62.71–65.13)	17.50 (16.96–18.05)	7.30 (6.98–7.61)	0.76 (0.68–0.83)
F-40	65.02 (63.92–66.12)	17.81 (17.32–18.31)	6.66 (6.36–6.95)	0.80 (0.72–0.88)
Charisma	51.51 (50.18–52.83)	16.88 (16.16–17.60)	8.49 (8.14–8.84)	0.73 (0.65–0.80)

**Table 4:** Fit parameters of exponential function (95% confidence interval in parentheses) for 2-mm layer.

	a	b	c	d
Control	64.34 (63.36–65.31)	17.30 (16.88–17.73)	7.37 (7.13–7.60)	0.68 (0.62–0.74)
C-5	71.64 (70.43–72.86)	16.49 (16.03–16.95)	7.54 (7.25–7.84)	0.65 (0.57–0.72)
C-10	66.51 (65.85–67.16)	13.54 (13.31–13.77)	7.45 (7.27–7.63)	0.60 (0.55–0.64)
C-20	61.89 (61.44–62.34)	10.12 (9.99–10.26)	7.73 (7.57–7.89)	0.44 (0.41–0.48)
C-40	58.62 (57.97–59.26)	8.26 (8.09–8.43)	7.38 (6.95–7.81)	0.34 (0.27–0.40)
F-5	65.75 (64.76–66.74)	17.47 (17.05–17.89)	8.05 (7.83–8.28)	0.64 (0.58–0.69)
F-10	66.51 (65.51–67.51)	17.61 (17.19–18.04)	7.67 (7.43–7.91)	0.69 (0.64–0.75)
F-20	66.96 (65.86–68.06)	18.17 (17.69–18.65)	7.77 (7.50–8.04)	0.74 (0.67–0.80)
F-40	68.75 (67.80–69.69)	16.85 (16.47–17.23)	7.64 (7.40–7.89)	0.71 (0.65–0.77)
Charisma	46.54 (45.62–47.45)	13.44 (12.98–13.91)	9.07 (8.77–9.37)	0.72 (0.66–0.77)

## Discussion

This study investigated the effect of two BG types that were added as functional fillers to experimental light-cured composites (0–40 wt%). The addition of both BG types reduced curing light transmittance through a 2-mm layer in a dose-dependent manner; however, a pronounced reduction of polymerization rate and DC was observed only for BG 45S5. In contrast, the customized F-containing BG had a negligible influence on polymerization. As the effect of BG on polymerization kinetic parameters and light transmittance was affected by BG amount and type, the null hypotheses were rejected.

A series of previous studies on experimental composites functionalized with BG 45S5 have evaluated their DC at a single time point after light-curing, namely 15 min^22^ or 24 h^17–19^, while one study additionally evaluated polymerization kinetics^20^. The present work complements these studies by introducing a series of composites functionalized with a customized low-Na F-containing BG^21,23^. Hence, the performance of this new composite series was compared to the previously investigated composite series containing BG 45S5, both series being based on a typical photo-curable Bis-GMA/TEGDMA (60:40 by weight) resin system. The present study results for the composite series containing BG 45S5 are in accordance with previous studies^17–20^, indicating that the addition of BG 45S5 can diminish both DC_5 min_ and R_max_. In contrast to the results for BG 45S5, the addition of customized F-containing BG showed a minor and practically negligible effect on the DC_5 min_ and R_max_.

In previous studies, the inhibitory effect of BG 45S5 on polymerization of experimental composites has been attributed to the ability of oxides on the surface of unsilanized BG particles for hindering the free radical-mediated polymerization reaction^17–20^. Although this phenomenon has not been extensively investigated in dental composites apart from the mentioned studies, other studies on epoxy^30^, polyester^31^, and methyl-methacrylate resins^32^ showed that electron-accepting oxides on surfaces of filler particles can inhibit free radical-mediated polymerization. Another explanation for the observed inhibitory effect may be the free radical-inactivating property of water on the surface of BG 45S5 particles which are hygroscopic due to high sodium content^11^. To minimize the uptake of atmospheric water, the BG fillers were originally packaged by the manufacturer in an inert gas environment and used as-received in the preparation of experimental composites. This procedure was performed under atmospheric conditions and allowed approximately 3 min for the hydrophilic BG particles to absorb atmospheric moisture. After composite mixing, the BG particles were embedded within hydrophobic resin and further risk of water uptake was negligible. Therefore, a limited uptake of atmospheric moisture during composite preparation cannot be ruled out, but excessive contamination of BG fillers with water seems unlikely.

All materials reached R_max_ within 2.4–3.4 s from the start of light-curing. These results agree with studies on commercial^33^ and experimental composites^20^, which showed that light-curing using contemporary curing units leads to polymerization rates reaching maximum values at very early phases of the reaction due to a high initiation rate coupled with a diminished termination rate due to autoacceleration^2^. As this increase in polymerization rate occurred at a short time-scale with regards to the time resolution of our spectrometer (2 spectra/s), the virtual absence of significant differences in R_max_ among the materials and layer thicknesses can be attributed to low statistical power due to high data scattering. Other studies with similar experimental setups have also identified R_max_ as being less discriminative than other polymerization kinetic parameters^33,34^.

The FTIR spectra were collected from several micrometres-thick layers of composite immediately adjacent to the ATR crystal, thus allowing localized DC measurements without the risk of oxygen inhibition at the bottom of different specimen thicknesses, namely 2 mm (clinically relevant) and 0.1 mm (thin film). The latter was used to minimize the effect of curing light attenuation on polymerization kinetics. Thin films are assumed to be sufficiently cured as they receive irradiance several orders of magnitude higher than layers of clinically relevant thickness^35^. Interestingly, the 0.1-mm specimens showed generally lower DC_5 min_ than the 2-mm specimens, which can be explained by more heat release in the thicker composite specimens, consequently improving the mobility of reactive species and allowing higher DC^36^. On the other hand, R_max_ was either unaffected by layer thickness (F-series) or higher for the 0.1-mm layer compared to the 2-mm layer due to a higher initiation rate in thin layers (C-series).

Because the mismatch in resin/filler refractive indices was higher for BG than reinforcing fillers, trans_ef_ was reduced with increasing BG amounts for both experimental composite series. Although trans_ef_ values differed widely among the experimental composites (4.9–18.5%), most of these values were significantly higher than that of the commercial composite (5.0%). It should be noted that the significant reduction in light transmittance due to increasing BG amounts in F-series (from 18.5 to 12.0%, for 0 and 40 wt% of BG, respectively) was not followed by a reduction in DC_5 min_ or R_max_. It has been demonstrated that for sufficiently translucent composites, moderate changes in light transmittance do not necessarily affect polymerization kinetics and final DC^27^. This was the case in the F-series which had high trans_ef_ values (2.4–3.4 times higher than the commercial reference composite), rendering the effect of light transmittance on polymerization kinetics indistinguishable.

In contrast to DC_5 min_ and R_max_ in the F-series being unaffected by trans_ef_ changes, the progressive reduction of trans_ef_ with higher BG amounts in the C-series was accompanied by lower DC_5 min_ and R_max_. The observed negative effect of BG on polymerization kinetics might have been exerted directly (through inhibition of free radical polymerization) or indirectly (through reduction in light transmittance). These two contributions can be differentiated by comparing the results obtained for 2-mm and 0.1-mm layers. Since in thin films the effect of light transmittance is negligible^35^, the fact that higher BG amounts diminished DC_5 min_ and R_max_ in a similar manner for both 2-mm and 0.1-mm layers indicates that the inhibitory effect of BG on polymerization kinetics was independent of the changes in light transmittance. In case that light transmittance was the primary cause for impaired polymerization, the reduction in DC_5 min_ and R_max_ would be more pronounced in the 2-mm layers than in the 0.1-mm layer.

R_max_ was higher in the 2-mm layers than in 0.1-mm layers only for the most translucent composite (Control); for other composites, R_max_ was either higher for the 0.1-mm layer (Charisma and C-series) or unaffected by layer thickness (F-series). The higher polymerization rate in 0.1-mm than in 2-mm layers is expected as the lower thickness allowed higher initiation rates at the specimen bottom. The discrepancy of polymerization being faster in the 2-mm layer observed for the Control composites is probably due to the joint effect of its high light transmittance and the heating effect of the thicker layer.

Refractive indices of various BG compositions can be theoretically predicted^11^ and tailored to ensure optimal translucency of composite materials. In practice, however, the variables affecting composite translucency (resin composition, filler composition, and filler particle size distribution) are usually determined by other requirements, e.g., for attaining appropriate viscosity and handling, desired reactivity, and final mechanical properties. Optimizing these parameters made the optical properties of the experimental composites in the present study a secondary outcome rather than a primary consideration. Nevertheless, light transmittance of the experimental composites was higher than that of the commercial reference composite, being comparable to values reported for highly translucent commercial bulk-fill composites^27^. Also, the customized F-containing BG allowed higher trans_ef_ for a given BG amount than BG 45S5 as adding CaF_2_ to the BG composition reduced its refractive index, allowing for better matching of resin/filler refractive indices^11^.

The experimental composites were formulated in accordance with previous studies, mimicking the composition of hybrid composite materials containing micro- and nano-sized fillers in a classic Bis-GMA/TEGDMA resin^17,20,21,23^. By increasing BG amounts from 0 to 40 wt% within a composite series, smaller reinforcing fillers (nano-sized silica and micro-sized barium glass) were replaced with larger BG particles, thereby decreasing the total filler surface area and consequently increasing resin mobility. This effect was identified in F-series in which BG-containing composites reached higher DC_5 min_ compared to the control composite. In the C-series, the effect of better resin mobility due to decreasing surface area by adding more BG 45S5 was observable only for BG amounts up to 10 wt%, whereas for higher BG loadings the effect was overcome by polymerization inhibition, resulting in reduced DC_5 min_.

The plots of polymerization rate versus DC show that polymerization reached maximum speed at early stages, for DC values below 15%. All composites from the F-series showed similar curves of polymerization rate versus DC, featuring the shape typical of multifunctional methacrylate polymerization, with a clearly defined ascending part (autoacceleration), turnover point (gelation point), and descending part (autodeceleration)^37^. In contrast, the shape of the polymerization rate versus DC curves for the C-series varied according to the BG amount; for higher BG amounts, the ascending part of the curves became less pronounced. For the composite with the highest amount of BG 45S5 (40 wt%), there was no discernible peak in the polymerization rate versus DC curves, as their polymerization rate steadily decreased as a function of DC. These deviations of polymerization rate versus DC curves from commonly observed shapes^38^ are likely related to the polymerization inhibition by BG 45S5.

The reduction of the parameter “a” with increasing BG amounts in the C-series indicates a lower extent of polymerization reached in the gel phase. The accompanying reduction of the parameter “b” shows that the polymerization rate in the gel phase of the C-series is also reduced as the BG amount increases. Assuming that the effect of curing light attenuation in 0.1-mm layers was negligible, the similar reduction pattern in “a” and “b” identified for both 0.1-mm and 2-mm layers supports our previous claims that the polymerization inhibition due to the addition of BG occurred independently of changes in light transmittance. The reduction of the parameter “c” (representing the extent of polymerization in the glass phase) with increasing BG amounts in the C-series was more pronounced in 0.1-mm specimens than in 2-mm specimens, while the corresponding reduction of the parameter “d” (representing the polymerization rate in the glass phase) was identified for both layer thicknesses. These results indicate that polymerization was hindered by higher BG amounts also in the glass phase.

In contrast to higher BG amounts diminishing the parameters “a” and “b” in the C-series, increasing the BG amount in the F-series led to a moderate increase in the parameter “a” and no significant effect on the parameter “b”. This suggests that the polymerization extent in the gel phase was slightly improved without an observable change in polymerization rate. The improvement in the polymerization extent is attributable to the aforementioned fact that increasing BG amounts reduced the filler particle surface area and improved molecular mobility. The parameter “c” in the F-series was reduced by higher BG amounts, indicating that the extent of reaction in the glass phase was somewhat lower, presumably due to a higher extent of reaction that occurred in the gel phase. The reaction rate in the glass phase of the F-series was not affected by the addition of BG, as suggested by similar values of the parameter “d”.

As this study investigated curing kinetics under in vitro conditions, it should be noted that composite polymerization and related phenomena (polymerization shrinkage and shrinkage stress) under clinically relevant conditions can differ from those recorded on freely-shrinking samples. The external constraint due to bonding to cavity walls has been shown to affect not only shrinkage stress development^39^ but also polymerization kinetics and final degree of conversion^40^.

## Conclusions

In this in vitro study, a customized low-Na F-containing bioactive glass showed favourable behaviour for being used as a functional filler in light-curing dental resin composites. Unlike bioactive glass 45S5, which led to a dose-dependent reduction in the rate and extent of polymerization, the customized low-Na F-containing bioactive glass showed a negligible influence on polymerization. The reduction in light transmittance of experimental composites due to the addition of the low-Na F-containing bioactive glass did not translate into impaired polymerization kinetics. Additionally, the comparison of polymerization kinetics between thin films (0.1 mm) and 2-mm thick layers revealed that polymerization inhibition identified for bioactive glass 45S5 was not mediated by an impaired light transmittance, indicating a direct effect on polymerization reaction.

## Data Availability

The datasets generated during and/or analysed during the current study are available from the corresponding author on reasonable request.
